# Proton radiotherapy for recurrent or metastatic sarcoma with palliative quad shot

**DOI:** 10.1002/cam4.3646

**Published:** 2021-06-04

**Authors:** Anna Lee, Jung J. Kang, Havah Bernstein, Kathryn E. Marqueen, Brian Neal, Ciara M. Kelly, Mark A. Dickson, Chiaojung Jillian Tsai, William Tap, Samuel Singer, Kaled Alektiar, Nancy Y. Lee

**Affiliations:** ^1^ Department of Radiation Oncology University of Texas MD Anderson Cancer Center Houston TX USA; ^2^ Department of Radiation Oncology Memorial Sloan Kettering Cancer Center New York NY USA; ^3^ ProCure Proton Therapy Center Somerset NJ USA; ^4^ Department of Medical Oncology Memorial Sloan Kettering Cancer Center New York NY USA; ^5^ Department of Medicine Weill Cornell Medical College New York NY USA; ^6^ Department of Surgical Oncology Memorial Sloan Kettering Cancer Center New York NY USA; ^7^ Department of Surgery Weill Cornell Medical College New York NY USA

**Keywords:** metastatic sarcoma, palliative treatment, proton therapy, quad shot regimen, recurrent sarcoma

## Abstract

Patients with previously treated, recurrent or metastatic sarcomas who have progressed on multiples lines of systemic therapy may have limited options for local control. We evaluated outcomes of palliative proton therapy with the quad shot regimen to unresectable disease for patients with recurrent and/or metastatic sarcoma. From 2014 to 2018, 28 patients with recurrent or metastatic sarcomas were treated to 40 total sites with palliative proton RT with quad shot (14.8 Gy/4 twice daily). Outcomes included toxicity, ability to receive further systemic therapy, and subjective palliative response. Univariate analysis was performed for local progression‐free survival (LPFS) and overall survival (OS). Of the 40 total sites, 25 (62.5%) received ≥3 cycles with median follow up of 12 months (IQR 4–19). The most common histologies were GIST (9; 22.5%) and leiomyosarcoma (7; 17.5%). A total of 27 (67.5%) sites were located in the abdomen or pelvis. Seventeen (42.5%) treatments involved concurrent systemic therapy and 13 (32.5%) patients received further systemic therapy following proton therapy. Overall subjective palliative response was 70%. Median LPFS was 11 months and 6‐month LPFS was 66.1%. On univariate analysis, receipt of four cycles of quad shot (HR 0.06, *p* = 0.02) and receipt of systemic therapy after completion of radiation therapy (HR 0.17, *p* = 0.02) were associated with improved LPFS. Three grade 3 acute toxicities were observed. The proton quad shot regimen serves as a feasible alternative for patients with previously treated, recurrent or metastatic sarcomas where overall treatment options may be limited.

## Graphical abstract

Patients with previously treated, recurrent or metastatic sarcomas who have progressed on multiple lines of systemic therapy have limited options for local control. We report on 28 patients treated to 40 sites with proton therapy using the quad shot regimen and found subjective palliative response rate of 70% and median LPFS of 11 months with little toxicity.

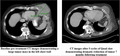

## 1 INTRODUCTION

Sarcomas are a rare and heterogeneous group of tumors that can arise from soft tissues or bones throughout the entire body. Due to their ability to arise from any anatomic site, proximity to critical structures can be challenging and limiting when local therapy is indicated. Surgical resection is the mainstay of therapy, and radiation therapy (RT) is typically utilized preoperatively or postoperatively. But for some patients with recurrent or metastatic sarcoma, surgery may not be possible and radiation therapy may be the only option for local disease management. There are no currently accepted standards for the most appropriate radiation regimen or expected outcomes and toxicities with definitive radiation therapy in the setting of unresectable recurrent or metastatic sarcomas.

The quad shot regimen was reported in the phase III RTOG 8502 trial and is a split course radiation treatment consisting of 3.7 Gy twice daily for 2 consecutive days at 2‐ to 4‐week intervals and was originally intended for treating pelvic malignancies in the palliative setting.^1^ Since then, this fractionation regimen has been utilized in other sites such as incurable head and neck cancers with overall tumor responses of 53%–77% and a 53%–85% improvement in symptom palliation.^2^, ^3^, ^4^ A total of four cycles of the quad shot regimen delivers an EQD2 (2 Gy equivalent dose) of 79 Gy (alpha‐beta 3), and hypofractionation may be of particular benefit to low alpha‐beta tumors like sarcoma.

The rapid adoption of proton therapy in many treatment sites has been in part due to its superior dosimetry, with steeper fall‐off of exit dose in critical areas. This property is especially advantageous for patients with recurrent tumors in previously irradiated areas as well as those near radiosensitive critical structures. A recent study utilizing proton therapy with the quad shot regimen for recurrent or metastatic head and neck cancer reported overall palliative response at 73% with no grade 3 or higher acute toxicities.^5^

To our knowledge, there are no reports on the utilization of quad shot radiation for local therapy in unresectable sarcoma. While there are data to support conventionally fractionated proton therapy in the primary management of sarcoma, the safety and feasibility of hypofractionated proton quad shot radiation therapy for unresectable sarcoma has not been established. To that end, we evaluated outcomes of palliative proton therapy with the quad shot regimen to unresectable disease for patients with recurrent or metastatic sarcoma.

## 2 MATERIALS AND METHODS

This retrospective study was independently reviewed and approved by the institutional review board. From 2014 to 2018, a total of 28 patients with recurrent or metastatic sarcomas outside the head and neck region were treated to 40 total sites with palliative proton RT at our center with the quad shot regimen. Those with spine or brain metastases were not included. Patients were determined to have unresectable tumors by a multidisciplinary team due to extent of disease, medical comorbidities, and/or distant metastatic burden.

The palliative proton quad shot regimen was administered at 3.7 Gy per fraction, assuming a relative biological effectiveness (RBE) value of 1.1. Each treatment cycle consisted of twice‐daily fractions administered over 2 consecutive days to a total of 14.8 Gy per cycle. Each cycle was repeated every 21–25 days in the absence of in‐field progression or significant acute toxicity. Treatment volumes were reviewed prior to each cycle and re‐planned to account for reduction in tumor volume for patients with significant response to therapy. Patients were treated with systemic therapy with cytotoxic or targeted agents at the discretion of the attending medical and radiation oncologist and was typically the planned strategy for those who received post‐RT chemotherapy.

Computed tomography (CT) imaging was used for patient simulation. Gross tumor volume included symptomatic gross disease and was identified through diagnostic imaging (including PET and MRI as available) and clinical examination when appropriate, then contoured onto the CT simulation images by the radiation oncologist. At the physician's discretion, a clinical target volume (CTV) specific to each patient was added to cover adjacent areas at high risk for microscopic tumor spread. The planning target volume (PTV) was created based on the CTV dependent upon available imaging guidance during treatment and setup uncertainty, which typically consisted of an additional 3‐ to 5‐mm margin. The PTV concept was utilized in lieu of robust planning due to limitations of the uniform scanning delivery technique (31 sites), and a desire to scalp dose around organs at risk (OAR) abutting the target. Nine sites were treated with single‐field, uniform dose pencil beam scanning. In all cases, the robustness of target coverage and OAR sparing was evaluated.

Palliative response was defined as subjective relief of presenting symptom(s) as determined through patient‐reported outcomes in clinical notes. Evaluation of the objective radiographic tumor response was performed at least 1 week after the last cycle of RT. Response evaluation criteria in solid tumors (RECIST) version 1.1 was used to determine objective response by two independent radiation oncologists (CR, complete response; PR, partial response; SD, stable disease; PD, progressive disease). Toxicity was assessed for all patients according to the Common Terminology Criteria for Adverse Events (version 5.0), with acute toxicity defined as any adverse event occurring during or within 3 months of completing palliative RT.

Follow‐up interval was defined as from the beginning of proton therapy until death or date of last contact. Analyzed factors included concurrent systemic therapy, Karnofsky performance status (KPS), tumor size, presenting symptoms, initial treatment, and total radiation dose. Outcomes of interest included toxicity, ability to receive further systemic therapy, subjective palliative response, and objective response. Local progression‐free survival (LPFS) was defined as survival from the start of proton therapy until radiographic progression of the treated lesion on surveillance imaging. Univariate Cox regression analysis was obtained for LPFS and overall survival (OS). Multivariate Cox regression for LPFS and OS was also performed and included the number of quad shot cycles, receipt of concurrent systemic therapy, and receipt of post‐RT systemic therapy.

## 3 RESULTS

Of the 40 total sites, 25 (62.5%) received ≥3 cycles. Median KPS was 90 at the time of first treatment. Median follow up was 12 months (IQR 4–19). The most common histologies treated were GIST (22.5%; *n* = 9) and leiomyosarcoma (17.5%; *n* = 7). Twenty‐seven (67.5%) sites were located in the abdomen or pelvis. Twenty‐six (66%) sites had been previously resected prior to recurrence and 5 (12.5%) sites had received prior RT to the same site or overlapping sites. The median prior RT dose was 58 Gy. Seventeen (42.5%) treatments involved concurrent systemic therapy and 13 (32.5%) patients received further systemic therapy following proton therapy. The most common systemic therapy agents utilized included dacarbazine (*n* = 3), nivolumab (*n* = 3), and imatinib (*n* = 3). Of the 17 sites treated with concurrent systemic therapy, 6 received chemotherapy, 10 received targeted therapy, and 1 received both (Table 1).

**TABLE 1:** Baseline clinical and treatment characteristics

	Results, *N* (%)
Total no. of patients	28
Total no. of treated lesions	40
Age, years, median (range)	61 (30–79)
Follow up, months, median (IQR)	12 (4–19)
Gender	
Men	17 (60.7)
Women	11 (39.3)
KPS, median (range)	90 (60–90)
Tumor size	
<10 cm	18 (45)
≥10 cm	22 (55)
Median, cm (range)	11 (3–27)
Histology	
GIST	9 (22.5)
Leiomyosarcoma	7 (17.5)
Myxofibrosarcoma	4 (10)
Spindle cell sarcoma	4 (10)
Dedifferentiated liposarcoma	4 (10)
Synovial sarcoma	2 (5)
Solitary fibrous tumor	2 (5)
Chondrosarcoma	2 (5)
Cardiac intimal sarcoma	2 (5)
Endometrial stromal sarcoma	2 (5)
Epithelial mesenchymal	1 (2.5)
Angiosarcoma	1 (2.5)
Anatomic distribution	
Abdomen	20 (50)
Pelvis	7 (17.5)
Lung	7 (17.5)
Trunk	4 (10)
Extremities	2 (5)
Concurrent systemic therapy	
Yes	17 (42.5)
No	23 (57.5)
Type of concurrent systemic therapy	
Chemotherapy	6 (35.3)
Targeted agent	10 (58.8)
Both	1 (5.9)
Prior RT to same site	
Yes	5 (12.5)
No	35 (87.5)
Median dose, Gy (range)	58 (50–112)
Prior surgery for primary disease	
Yes	26 (65)
No	14 (35)
Cycles of quad shot	
1 (14.8 Gy)	8 (20)
2 (29.6 Gy)	7 (17.5)
3 (44.4 Gy)	17 (42.5)
4 (59.2 Gy)	8 (20)
Proton technique	
Uniform scanning	31
Pencil beam scanning	9
Post‐RT systemic therapy	
Yes	13 (32.5)
No	27 (67.5)

**FIGURE 1:**
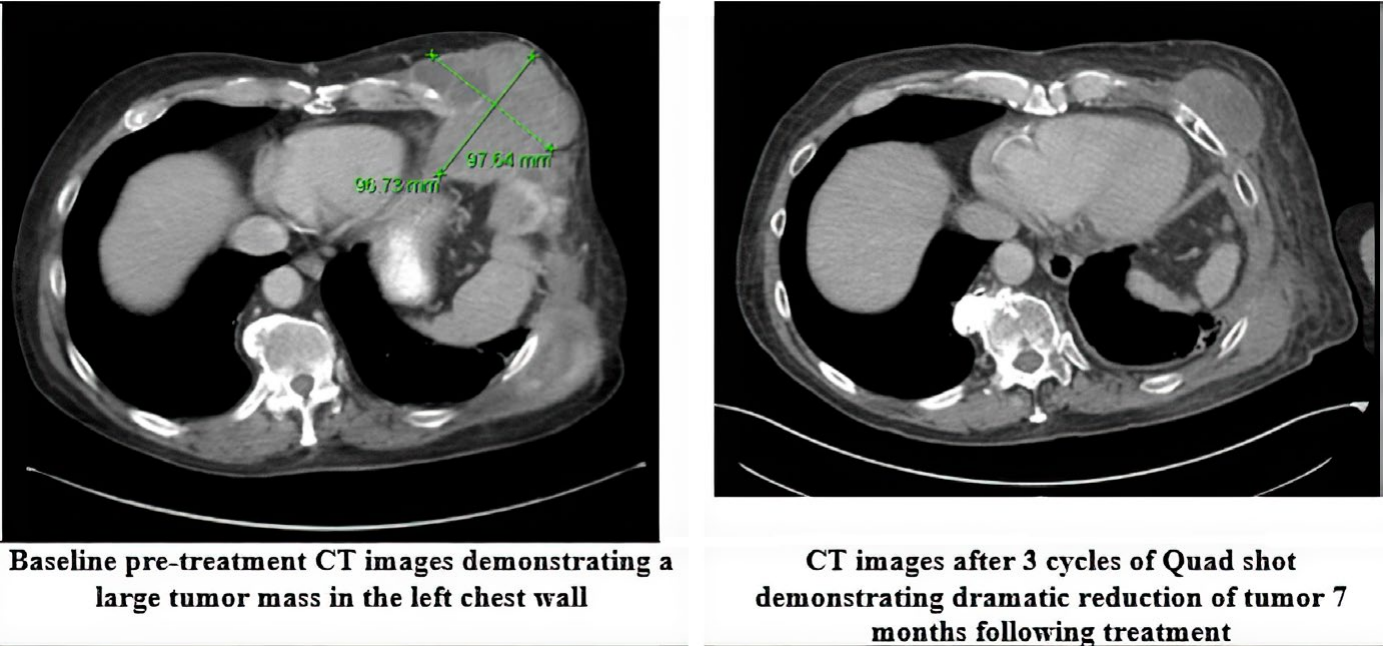
A 78‐year old man with high‐grade myxofibrosarcoma of the abdominal wall initially resected now with multiple recurrences with a total of six total surgical resections who received treatment to multiple sites including the chest wall lesion (pictured here) with partial response after three cycles of quad shot

Overall subjective palliative response was 70%. The most common presenting symptom was pain (77.5%, *n* = 31), which improved in 67.7% (*n* = 21) of cases. Of the 35 targets with post‐treatment imaging, overall objective response showed RECIST SD (32.5%, *n* = 13) or RECIST PR (15%, *n* = 6) in approximately half of all lesions (Table 2). There were no patients who had a CR and the rest had PD (40%, *n* = 16). Figure 1 shows an example of a patient with partial response after three cycles of quad shot to a left chest wall mass. A third of patients (32.5%, *n* = 13) received further systemic therapy following treatment with proton therapy.

**TABLE 2:** Presenting symptoms and palliative response by number of completed quad shot cycles

	Treatments, *N* (%)	Palliative response, *N* (%)
Presenting symptoms and palliative response		
Pain	31 (77.5)	21 (67.7)
Growth	8 (20)	5 (62.5)
Lymphedema	4 (10)	3 (75)
Neuropathy	1 (2.5)	1 (100)
Dyspnea	1 (2.5)	1 (100)
Hemoptysis	1 (2.5)	–
Objective radiographic tumor response		
Progression of disease	16 (40)	
Stable disease	13 (32.5)	
Partial response	6 (15)	
Complete response	0 (0)	
No post‐RT imaging	5 (12.8)	

No grade 4–5 acute toxicities were observed in our study. There were seven grade 1 toxicities, six grade 2 toxicities, and three grade 3 toxicities. The most common adverse effect was fatigue followed by nausea. Of the five patients who had prior RT, there was one grade 1 fatigue. Grade 3 abdominal infection, diarrhea, and colonic obstruction were observed. None of these patients received concurrent systemic therapy (Table 3).

**TABLE 3:** Acute toxicities observed in patients after quad shot (CTCAE)

	Grade 1, No.	Grade 2, No.	Grade 3, No.
Fatigue	4	2	–
Dyspnea	–	1	–
Abdominal infection	–	–	1
Duodenal fistula	1	–	–
Nausea	–	3	–
Cough	1	–	–
Vomiting	1	–	–
Diarrhea	–	–	1
Colonic obstruction	–	–	1

Abbreviation: CTCAE, Common Terminology Criteria for Adverse Events.

On Kaplan–Meier time‐to‐event analysis, median LPFS was 11 months and 6‐month LPFS was 66.1%. Median OS was 12 months and 6‐month OS was 65%. On univariate Cox regression analysis, receipt of four cycles of quad shot (HR 0.06, 95% CI 0.01–0.65, *p* = 0.02) and receipt of systemic therapy after completion of radiation therapy (HR 0.17, 95% CI 0.04–0.74, *p* = 0.02) were associated with improved LPFS. Similarly, receipt of at least three cycles (HR 0.14–0.17, *p* = 0.01) compared to one cycle of quad shot and receipt of further systemic therapy (HR 0.34, 95% CI 0.14–0.79, *p* = 0.01) were associated with improved OS. Tumor size ≥10 cm (HR 3.65, 95% CI 1.64–8.13, *p* = 0.01) was associated with worse survival (Table 4). To ascertain the effects of treatment on outcomes, multivariate Cox regression was conducted showing four cycles of quad shot (HR 0.05, *p* = 0.02) and post‐RT systemic therapy (HR 0.10, *p* = 0.01) were associated with improved LPFS while three to four cycles of quad shot (HR 0.10–0.18, *p* = 0.01) and post‐RT systemic therapy (HR 0.23, *p* = 0.01) were associated with improved OS (Table 5).

**TABLE 4:** Univariate Cox regression analysis of local progression‐free survival (LPFS) and overall survival (OS)

	LPFS		OS	
	HR (95% CI)	*p*‐value	HR (95% CI)	*p*‐value
KPS				
<80	1		1	
≥80	0.24 (0.03–2.11)	0.20	0.36 (0.10–1.28)	0.12
Gender				
Male	1		1	
Female	1.26 (0.43–3.74)	0.68	1.70 (0.82–3.55)	0.16
Tumor size				
<10 cm	1		1	
≥10 cm	2.43 (0.87–6.77)	0.09	3.65 (1.64–8.13)	0.01
Age at quad shot start (continuous variable)	0.98 (0.93–1.03)	0.33	0.96 (0.93–0.99)	0.03
Concurrent systemic therapy				
No	1		1	
Yes	0.75 (0.28–2.00)	0.57	0.55 (0.26–1.17)	0.12
Prior RT to same site				
No	1		1	
Yes	0.86 (0.24–3.03)	0.81	0.44 (0.13–1.46)	0.18
Cycles of quad shot				
1	1		1	
2	0.81 (0.12–5.38)	0.82	0.56 (0.19–1.65)	0.29
3	0.19 (0.03–1.31)	0.09	0.17 (0.06–0.51)	0.01
4	0.06 (0.01–0.65)	0.02	0.14 (0.04–0.50)	0.01
Post‐RT systemic therapy				
No	1		1	
Yes	0.17 (0.04–0.74)	0.02	0.34 (0.14–0.79)	0.01

**TABLE 5:** Multivariate Cox regression analysis of local progression‐free survival (LPFS) and overall survival (OS)

	LPFS		OS	
	HR (95% CI)	*p*‐value	HR (95% CI)	*p*‐value
Concurrent systemic therapy				
No	1		1	
Yes	0.35 (0.09–1.35)	0.13	0.64 (0.28–1.45)	0.28
Cycles of quad shot				
1	1		1	
2	1.73 (0.27–11.02)	0.56	0.86 (0.28–2.59)	0.78
3	0.23 (0.03–1.90)	0.17	0.18 (0.06–0.55)	0.01
4	0.05 (0.00–0.63)	0.02	0.10 (0.03–0.38)	0.01
Post‐RT systemic therapy				
No	1		1	
Yes	0.10 (0.02–0.51)	0.01	0.23 (0.09–0.61)	0.01

## 4 DISCUSSION

In this single institutional study, we found that quad shot is an effective local therapy for gross unresectable disease in patients with recurrent or metastatic sarcoma. Patients experienced an overall subjective palliative response rate of 70%, and 48% of lesions achieved objective radiographic response with interval stability or shrinkage on imaging. The LPFS was 66% at 6 months, and nearly one third of patients were able to move forward to systemic therapy after treatment. Receipt of more cycles of quad shot and receipt of concurrent chemotherapy were associated with improved LPFS and overall survival.

Patients with recurrent or metastatic sarcoma and gross unresectable disease would be predicted to have dismal prognosis, with an objective response rate to first‐line chemotherapy of <20% and a median duration of only 6 months.^6^ However, most patients in our cohort had clinical and/or radiographic response, and a substantial proportion of patients attained enough benefit from therapy to move on to life‐sustaining systemic therapies after radiation. There is limited data with respect to local control rates following radiation therapy in this clinical scenario. One study of proton reirradiation for recurrent and secondary soft tissue sarcomas found 12 of 23 patients experienced local failure at a median of 10 months,^7^ which is similar to the median LPFS of the present study of 11 months. When stratified by resection at recurrence, there was no difference in incidence of local failure with versus without resection.

The management of recurrent or metastatic sarcoma can be challenging as the clinical presentations are heterogeneous and complex. Per the National Comprehensive Cancer Network (NCCN), guidelines are intentionally nonspecific about treatment options for this variegated group of patients.^8^ Radiotherapy is rarely considered for first‐line therapy as patients typically have multiple lesions and systemic therapy can treat primary and disseminated lesions. However, studies have shown effectiveness in the palliation of symptoms with radiation. A retrospective analysis of patients treated with a hypofractionated regimen of 39 Gy in 13 fractions of 3 Gy/day found durable pain control in 12 of 15 cases treated for gross metastases. At median follow up of about 6 months, the only acute side effect was grade 1 dermatitis.^9^ A larger study of 114 soft tissue and 23 bone sarcomas treated for palliative intent found symptomatic improvement in 70% of soft tissue and 55% of bone sarcoma patients with response rate increasing with biological effective dose (BED) of 50 Gy_4_ (*α*/*β* = 4 for tumor),^10^ which is similar to the response rate in the present study.

Sarcomas are commonly regarded as relatively radioresistant, with doses of 60–70 Gy in 2 Gy per fraction required in the postoperative setting to control subclinical disease.^11^ Proton therapy lends itself well to treating large unresectable tumors, especially when surrounded by radiosensitive organs such as the kidneys, liver, bowel, and spinal cord. The steep dose fall‐off from the Bragg peak phenomenon allows sparing of normal tissues distal to the target. Its benefit is particularly evident in retroperitoneal and intra‐abdominal sarcomas where a comparison study of proton therapy to intensity‐modulated radiation therapy and 3D conformal photon radiation therapy found integral dose was more than 60% lower in patients treated with protons,^12^ which is expected to translate into decreased toxicity and improved therapeutic index. The clear dosimetric advantages to proton therapy particularly in the abdomen and pelvis have been described in multiple studies.^13^, ^14^, ^15^

In the current analysis, proton therapy was well tolerated with no grade 4 or higher toxicities with the most common adverse effect being fatigue and nausea. Three grade 3 toxicities of abdominal infection, diarrhea, and colonic obstruction were observed. Upon close examination, none of these patients had received concurrent systemic therapy and the cause of toxicity was considered to be multifactorial with some component of tumor effect. In one of few retrospective studies in the literature comparing proton to photon therapy in abdominal malignancies, proton therapy was associated with decreased risk of radiation‐induced liver disease while locoregional control remained comparable with photon therapy.^16^ Furthermore, it is promising that the administration of concurrent systemic therapy was safe in the current analysis.

The quad shot schedule is a hypofractionated regimen that allows target revision prior to each cycle to account for changes in tumor volume. The adaptive re‐planning allows even more sparing of normal tissue as anatomical shifts may occur with decrease in tumor size. One of the biological characteristics of sarcomas is the relatively low α/β ratio, which justifies the use of larger fraction sizes in order to achieve cell kill. A large series of heavily pretreated sarcoma patients treated for pulmonary metastases with stereotactic body radiation therapy found 1‐year local control of 94% and 2‐year local control of 86%. Radiation was administered to 50 Gy in 4–5 fractions.^17^ A study calculating the biological effectiveness of the quad shot regimen found 4–5 cycles is comparable to that from protracted definitive RT regimens.^18^ Thus, in the palliative setting, quad shot is especially favorable as it meets all the major goals of therapy: attaining local tumor control, providing symptom palliation, an abbreviated treatment course, and minimal toxicity.

The combination of advanced technology can further enhance the efficacy of the quad shot. Our group previously reviewed 26 patients with recurrent or metastatic head and neck cancer treated with quad shot dosing using proton therapy and found those who received three or more cycles had higher palliative response rate at 88% compared to 35% after one cycle and 68% after two cycles.^5^ More quad shot cycles were significantly associated with both PFS and OS, which was also observed in this analysis with four cycles associated with improved LPFS (HR 0.06, *p* = 0.02) and three or more cycles associated with improved OS (HR 0.14–0.17, *p* = 0.01). These results were upheld in the multivariate analysis. Another recent analysis from our group studying quad shot as last‐line local treatment for previously irradiated head and neck cancer found an overall palliative response rate of 66% with 1‐year LPFS of 17.7% and 1‐year OS of 25.3%. On multivariate analysis, proton therapy (compared to photon therapy), KPS > 70, receipt of 3–4 cycles of quad shot, and presence of palliative response were associated with improved LPFS and OS.^19^ Altogether, these studies support the utilization of the quad shot regimen with proton therapy in the palliative setting.

As with any other retrospective analysis, there are limitations and associated biases related to this type of review. Toxicity was low; however, many did not live long enough to develop late radiation toxicity. The cost of proton therapy is an ongoing source of contention, with proton therapy administered only to those with the resources to access a proton facility. This may introduce selection bias into any proton study, but costs and geographic barriers are expected to decrease as the number of operational centers increases. The potential value of this modality in the palliative setting has been suggested by institutional studies but needs widespread validation. As in any sarcoma analysis, another limitation of this study was patient heterogeneity.

## 5 CONCLUSIONS

In conclusion, the quad shot regimen with proton therapy demonstrates favorable palliative response and toxicity profile. Even in the setting of gross disease, 33% of patients went on to receive further systemic therapy, which was associated with improved survival. The results reported offer encouraging data for a feasible alternative for patients with previously treated, recurrent, or metastatic sarcomas where overall treatment options may be limited.

## CONFLICT OF INTEREST

NL‐Consultant for Pfizer, Merck, Merck Serono; research funding from Merck, Pfizer, and Astra Zeneca.

## Data Availability

The data that support the findings of this study are available from the corresponding author upon reasonable request.
